# Factors associated with change in exacerbation frequency in COPD

**DOI:** 10.1186/1465-9921-14-79

**Published:** 2013-07-30

**Authors:** Gavin C Donaldson, Hanna Müllerova, Nicholas Locantore, John R Hurst, Peter MA Calverley, Jorgen Vestbo, Antonio Anzueto, Jadwiga A Wedzicha

**Affiliations:** 1 Centre for Respiratory Medicine, UCL Medical School, Royal Free Campus, Rowland Hill Street Hampstead, London NW3 2PF, UK; 2 GlaxoSmithKline R&D, Building 9, Iron Bridge Road, Stockley Park West, Uxbridge, Middlesex UB11 1BT, UK; 3 GlaxoSmithKline, Research Triangle Park, NC, USA; 4 Centre of Inflammation and Tissue Repair, University College London, London, UK; 5 School of Ageing and Chronic Disease, University Hospital Aintree, Lower Lane, Liverpool L9 7AL, UK; 6 Department of Respiratory Medicine, Odense University Hospital and University of Southern Denmark, Odense, Denmark; 7 Respiratory Research Group, School of Translational Medicine, Manchester Academic Health Sciences Centre, University of Manchester, Manchester, UK; 8 Pulmonary Section, University of Texas Health Science Center, and South Texas Veterans Health Care System, San Antonio, TX, USA

**Keywords:** COPD, Exacerbations, Exacerbation frequency, Exacerbation phenotype

## Abstract

**Background:**

Patients with chronic obstructive pulmonary disease (COPD) can be categorized as having frequent (FE) or infrequent (IE) exacerbations depending on whether they respectively experience two or more, or one or zero exacerbations per year. Although most patients do not change category from year to year, some will, and the factors associated with this behaviour have not been examined.

**Methods:**

1832 patients completing two year follow-up in the Evaluation of COPD Longitudinally to Identify Predictive Surrogate End-points (ECLIPSE) study were examined at baseline and then yearly. Exacerbations were defined by health care utilisation. Patient characteristics compared between those patients who did or did not change exacerbation category from year 1 to year 2.

**Findings:**

Between years 1 and 2, 221 patients (17%) changed from IE to FE and 210 patients (39%) from FE to IE. More severe disease was associated with changing from IE to FE and less severe disease from FE to IE. Over the preceding year, small falls in FEV_1_ and 6-minute walking distance were associated with changing from IE to FE, and small falls in platelet count associated with changing from FE to IE.

**Conclusion:**

No parameter clearly predicts an imminent change in exacerbation frequency category.

**Trial registration:**

SCO104960, clinicaltrials.gov identifier NCT00292552

## Background

The natural history of chronic obstructive pulmonary disease (COPD) is punctuated by exacerbations defined as episodes of acute symptomatic deterioration that may warrant a change in treatment [1,2]. Frequent exacerbations are associated with reduced quality of life [3], increased airway inflammation [4], accelerated lung function decline [5] and increase mortality [6]. Patients with frequent exacerbations make more visits to their medical practitioner, require more medication, experience more hospital admissions [7] and consequently impose a greater financial burden on health services [8,9].

The number of exacerbations a patient experiences over a year is very strongly associated with their frequency in the previous year [3,7]. This stability in exacerbation frequency means that patient recall of events in the previous year can be used to categorize patients as having frequent (FE) or infrequent (IE) exacerbations during the subsequent year [7,10]. These categories are now recognised as important COPD patient phenotypes that influence the clinical management of the patient [11]. Although exacerbation frequency is relatively stable in most patients, some year on year variation exists and the factors associated with any change in category have not yet been investigated. Therefore we analysed the data from the “Evaluation of COPD longitudinally to identify predictive surrogate endpoints (ECLIPSE)” study to determine whether patients kept or changed their IE or FE category. Previously, three groups of 0, 1 and 2+ exacerbations per a year have been described [7] but interpretation of the nine potential transitions would be complex and less clinically relevant. In this study, we have also examined a slightly smaller group of 1832 patients who completed two years of observation rather than the 2138 patients who were enrolled into the ECLIPSE study [7] since the exacerbation frequency measured over both years 1 and 2 was needed to identify patients who did or did not changed IE and FE categories.

Additionally we tried to establish whether specific factors were associated with a change in exacerbation frequency as such data would be useful in identifying patients with an increased risk of a worsening exacerbation frequency and potentially suggest ways to prevent this clinically important change. Some of the results of this study have been previously reported in the form of an abstract [12].

## Methods

### Study design and patients

All patients provided written informed consent and the study was approved by the relevant ethics and review boards ([13]; see online supplement). Eligible patients were aged between 40 and 75 years, with a 10 or more pack-year smoking history, a post-bronchodilator forced expiratory volume in 1 second (FEV_1_) of less than 80% predicted from age, height and sex [14] and a ratio of FEV_1_ to forced vital capacity (FVC) of 0.7 or less.

**Figure 1:**
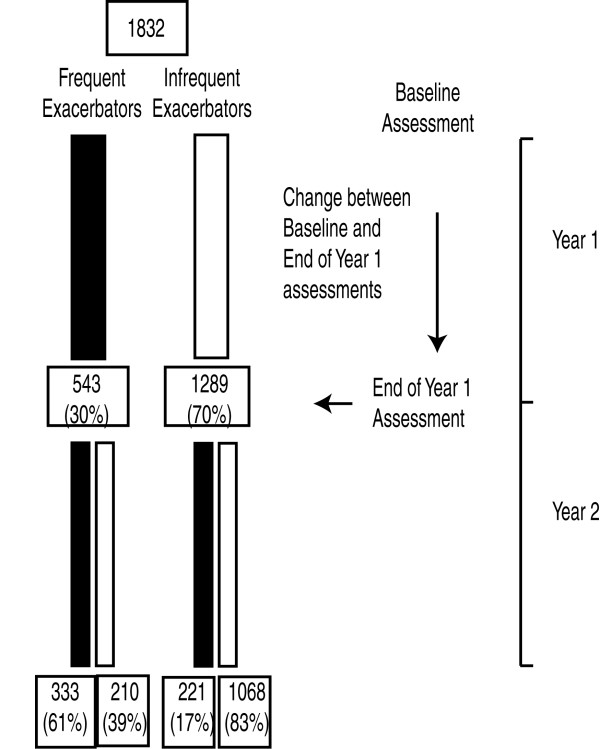
Timing of assessment and patient exacerbation categorization during the initial two years of the ECLIPSE study.

All patients completing at least 2 years of observation are included in this study. Patients underwent comprehensive medical reviews at baseline and then at the end of each 12 months of observation (see Figure 1). Data recorded at the 3, 6 and 18 month visits have not been analysed in this study as data on some patient characteristics were not available at these time points. Patients underwent standard spirometry testing after the administration of 400 μg of inhaled albuterol, completed a modified Medical Research Council dyspnoea scale (mMRC) and a St. Georges Respiratory Questionnaire (SGRQ). Co-morbidities were assessed from questions in the American Thoracic Society–Division of Lung Diseases (ATS-DLD) questionnaire and symptoms of depression by the Center for Epidemiologic Studies of Depression (CES-D) questionnaire. A venous blood sample was taken and a medical history including start/stop dates of medications and smoking history recorded. The patients underwent a six minute walking test (6MWD) according to ATS guidelines [15], with distance expressed as a percentage of predicted [16] which was used in calculating the patient’s BODE index [17]. Patients also underwent computed tomographic (CT) scanning, at baseline and year 1, to evaluate the severity of emphysema as the percentage of low attenuation areas (LAA%) [18].

Blood samples were dispatched immediately after each visit for a full-blood count, and serum and plasma samples stored at -80°C until assayed for fibrinogen, creactive protein (CRP), surfactant protein D (SP-D) and pulmonary activation regulated chemokine (PARC) [19]. Any samples with values below the lower limit of quantification were assigned a value that was half of the assay lower limit.

Exacerbations were defined by health care utilization requiring the prescription of antibiotics and/or oral corticosteroids by the patient’s primary care clinician or study personnel, or hospitalisation for an exacerbation. To ensure capture of these events patients were asked at clinic visits and during monthly telephone calls about visits to health care professionals and change in medications. The end of an exacerbation was based on investigator judgement, and no new exacerbation could start before resolution of an existing exacerbation. Frequent exacerbations were defined as 2 or more per year, and infrequent exacerbations as 1 or zero per year.

### Statistical analysis

Patients were categorized according to their exacerbation frequency assessed over year 1 of follow-up; whether they changed category was assessed using year 2 of follow-up. No data on how many exacerbations the patient recalled experiencing in year prior to recruitment was used in this analysis. Cross-sectional patient characteristics obtained at the year 1 assessment were examined in relation to those who did or did not change category, rather than the baseline assessment, as this was closest in time to the point of category change. “Longitudinal” characteristics were calculated as the difference between baseline and the year 1 assessment - that is, over the year preceding the point of change in exacerbation frequency (see Figure 1).

Means and standard deviations are reported for the continuous variables, with frequencies reported for the categorical variables. For tests between exacerbation frequency groups, an analysis of variance (ANOVA) for continuous variables or chi-square test for categorical variables was used. Due to skewed distributions, blood and plasma biomarkers were summarized using medians and inter-quartile ranges (IQR). Comparisons of their distributions across exacerbation frequency groups used a Kruskal-Wallis test.

## Results

### Change in exacerbation category

1832 patients completed at least 2 years of follow-up and had data available for this analysis.

543 of the 1832 patients (30%) had frequent exacerbations and 1289 (70%) infrequent exacerbations at the end of year 1 of follow-up. Between years 1 and 2, 431 of the 1832 patients (24%) changed category, 221 (17%) of the IE patients changed category to FE compared to 210 (39%) of the FE patients who changed to IE (P<0.001).

Table 1 shows the demographic characteristics of all 1832 patients recorded at the year 1 assessment, and after division into infrequent (IE) and frequent exacerbator (FE) groups by their observed exacerbation frequency over year 1.

**Table 1:** **Patient characteristics at the year 1 assessment**: **data is presented for all patients and sub**-**divided by exacerbation category in year 1 of follow**-**up**

	**All COPD** (**n**=**1832**)	**FE (n=543)**	**IE** (**n**=**1289**)	**p**-**value FE v IE**
Age (years)	64 (7)	64 (7)	64 (7)	0.751
Females (%)	35%	41%	32%	<0.001
BMI (kg/m2)	27 (6)	26 (6)	27 (6)	0.006
Active smoker (%)	36%	33%	37%	0.113
*FEV1 (L)	1.37 (0.55)	1.14 (0.45)	1.47 (0.55)	<0.001
*FEV1 %predicted	49 (16)	43 (16)	52 (16)	<0.001
*FEV1/FVC %	45 (12)	41 (12)	46 (12)	<0.001
6-minute walk distance (m)	385 (123)	350 (119)	399 (122)	<0.001
%predicted 6MWD	74 (24)	69 (23)	77 (23)	<0.001
Blood oxygen saturation (%)	94.7 (2.9)	94.2 (3.1)	94.9 (2.8)	<0.001
Emphysema LAA% (-950HU)	18 (12)	20 (13)	17 (12)	<0.001
mMRC Dyspnoea Score	1.7 (1.1)	2.0 (1.1)	1.5 (1.0)	<0.001
SGRQ-C Total Score	47 (21)	57 (18)	43 (21)	<0.001
BODE Index	3.0 (2.1)	3.9 (2.2)	2.7 (2.0)	<0.001
White blood cells (10^9/L)	7.6 (2.2)	8.1 (2.4)	7.4 (2.0)	<0.001
Platelets (10^9/L)	266 (71)	276 (75)	261 (69)	0.002
Fibrinogen (mg/dL)	436 [383–504]	468 [405–535]	426 [376–492]	<0.001
CRP (mg/dL)	3.3 [1.5-7.3]	4.1 [1.8-9.1]	3.1 [1.4-6.6]	<0.001
SP-D (ng/mL)	119 [87–163]	115 [86–159]	121 [87–166]	0.294
PARC/CCL-18 (ng/mL)	112 [87–145]	119 [92–154]	111 [85–141]	0.001
Neutrophils (10^9/L)	5.0 (1.9)	5.4 (2.2)	4.8 (1.8)	<0.001
Treated with ICSL %	71%	81%	66%	0.159
Treated with LAMA %	63%	68%	60%	<0.001
Treated with ICS %	17%	15%	17%	0.336
Treated with LABA %	14%	14%	15%	0.813
Treated with Oxygen %	10%	16%	6%	<0.001
PPPY COPD Hosp. Year 1	0.20	0.55	0.05	<0.001
PPPY OCS only exbs Y1	0.13	0.35	0.04	<0.001
Diabetes	10%	8%	10%	0.287
Cardiovascular (excl. HTN)	33%	32%	33%	0.868
Reflux/heartburn	26%	33%	23%	<0.001
Anxiety	16%	21%	14%	<0.001
Depression symptoms	25%	33%	21%	<0.001

P-values are for comparisons between patients with Frequent (FE) and Infrequent exacerbations (IE). Mean (SD) or Median [25% - 75% percentile].

### Change in exacerbation frequency

**Figure 2:**
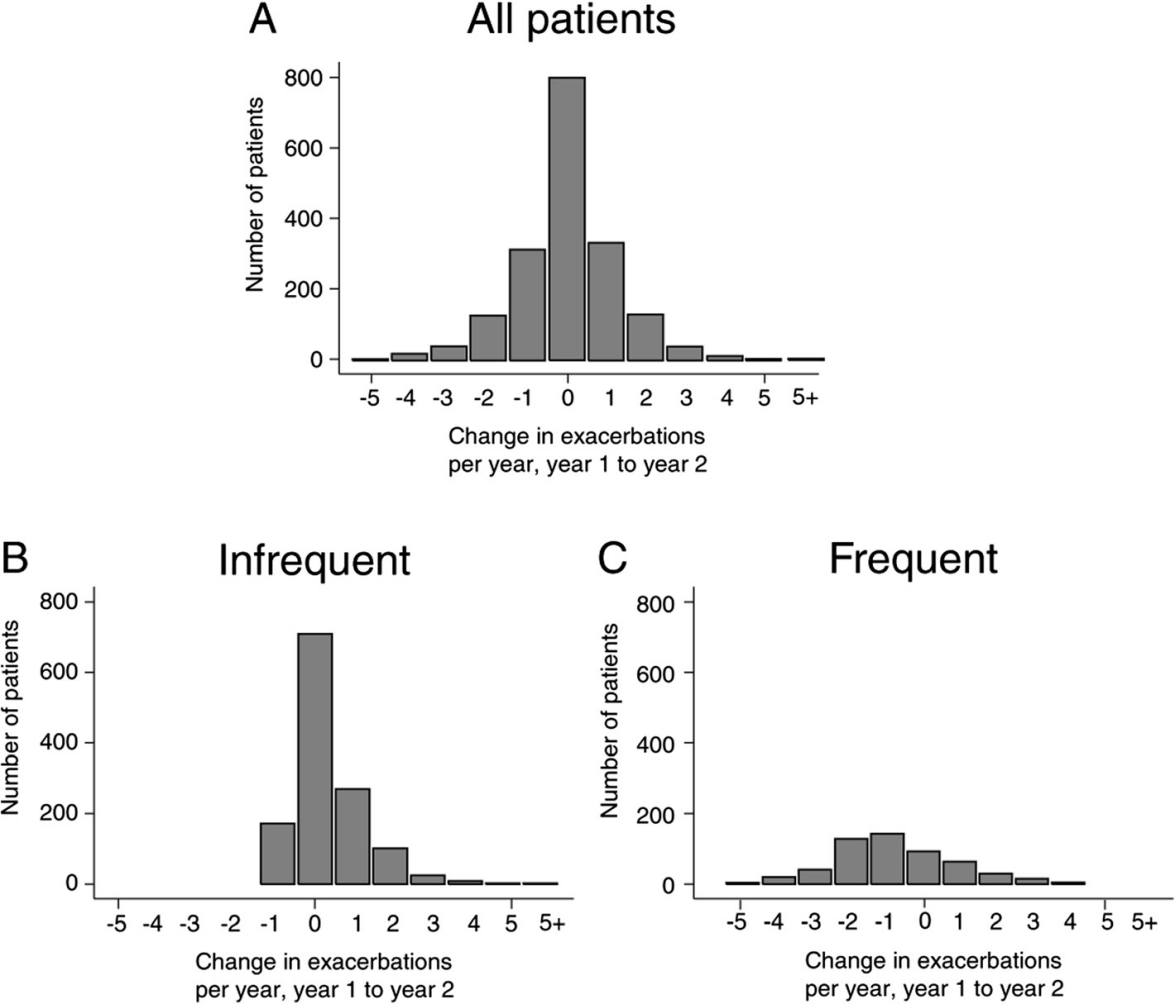
**Distribution of exacerbation frequency change.** All COPD patients **(A)** and in patients classified as IE **(B)** and FE **(C)** in year 1.

Figure 2A shows a histogram for the change in exacerbation frequency between years 1 and 2 of the 1832 patients. 44% of the patients had an identical exacerbation frequency in years 1 and 2, and for those patients whose exacerbation frequency did change, the magnitude and direct of the changes were evenly distributed around zero. This was consistent with the finding that the number of exacerbations per patient per year (PPPY) was 1.17 in both year 1 and year 2. However, when sub-divided into IE and FE groups, exacerbation frequency was seen to increased from 0.35 PPPY in year 1 to 0.70 PPPY in year 2, while in the FE group, it fell from 3.10 PPPY in year 1 to 2.30 PPPY in year 2. Figures 2B and 2C show that the distribution of these changes was not symmetrical. In the IE group, the distribution was skewed because exacerbation frequency could not fall by more than 1 exacerbation per year. In the FE group, without such restriction, there was a more even distribution but still many more patients in the -1 and -2 category than in the +1 or +2 category.

### Patient characteristics associated with IE to FE, and FE to IE category changes

Table 2 shows the cross-sectional characteristics of patients at the year 1 assessment, divided into those who did or did not change exacerbation category between years 1 and 2. Patients who changed from IE to FE had worse, and those changing from FE to IE better baseline post-bronchodilator FEV_1_, FEV_1_% predicted, mMRC, SGRQ-C, BODE, anxiety and hospitalised exacerbations per patient per year than those who remained in the respective categories. However, some patient characteristics were unique to the direction of change. Patients changing from IE to FE were more likely to be female, have a lower BMI, poorer FEV_1_/FVC ratio, less diabetes, more symptoms of depression and more emphysema (LAA%), but these factors were not different in those who remained FE compared to those who changed from FE to IE. Similarly, for the FE to IE transition, a greater 6MWD and lower systemic inflammation (CRP, Fibrinogen, PARC) were significant factors not seen in IE patients changing to FE.

**Table 2:** Patient characteristics at year 1 assessment split by exacerbation phenotype over study years 1 and 2

**Year 1 Category**	**Year 1 IE** (**n**=**1289**)			**Year 1 FE** (**n**=**543**)		
**Year 2 Category**	**Stayed as IE** (**n**=**1068**)	**Change to FE** (**n**=**221**)	**p**-**value**	**Stayed as FE** (**n**=**333**)	**Change to IE** (**n**=**210**)	**p**-**value**
**Characteristics at Year 1 Assessment**						
Age (years)	64 (7)	64 (7)	0.496	64 (7)	65 (7)	0.508
Females (%)	31%	41%	0.002	42%	40%	0.561
BMI (kg/m2)	27 (6)	26 (5)	0.007	26 (6)	26 (5)	0.918
Active smoker (%)	37%	35%	0.646	33%	32%	0.776
*FEV1 (L)	1.52 (0.56)	1.23 (0.45)	<0.001	1.09 (0.41)	1.23 (0.50)	<0.001
*FEV1 %predicted	53 (16)	45 (15)	<0.001	41 (15)	46 (16)	0.001
*FEV1/FVC %	47 (12)	43 (11)	<0.001	41 (11)	43 (12)	0.098
6-minute walk distance (m)	402 (124)	388 (114)	0.125	339 (125)	368 (107)	0.006
%predicted 6MWD	77 (23)	76 (22)	0.480	66 (24)	73 (21)	0.002
Blood oxygen saturation (%)	95.0 (2.8)	94.4 (2.7)	0.012	93.9 (3.2)	94.5 (2.7)	0.031
Emphysema LAA% (-950HU)	16 (12)	20 (13)	<0.001	21 (13)	19 (13)	0.126
mMRC Dyspnoea Score	1.5 (1.0)	1.7 (1.1)	0.004	2.1 (1.0)	1.8 (1.1)	<0.001
SGRQ Total Score	41 (18)	47 (19)	<0.001	57 (16)	50 (16)	<0.001
BODE Index	2.5 (1.9)	3.3 (2.0)	<0.001	4.2 (2.2)	3.4 (2.2)	<0.001
White blood cells (10^9/L)	7.4 (2.0)	7.5 (2.2)	0.926	8.2 (2.5)	7.9 (2.1)	0.082
Neutrophils, (10^9/L)	4.8 (1.7)	4.9 (2.1)	0.601	5.6 (2.4)	5.2 (1.9)	0.084
Platelets (10^9/L)	261 (70)	263 (69)	0.713	281 (77)	268 (70)	0.055
Fibrinogen (mg/dL)	423 [376–490]	438 [380–502]	0.092	480 [416–543]	444 [388–508]	<0.001
CRP (mg/dL)	3.0 [1.4-6.5]	3.4 [1.6-7.6]	0.071	4.7 [2.1-10.4]	3.1 [1.6-6.9]	0.002
SP-D (ng/mL)	121 [86–167]	125 [89–162]	0.625	116 [85–162]	114 [88–157]	0.651
PARC/CCL-18 (ng/mL)	111 [86–141]	110 [83–141]	0.943	121 [94–162]	113 [87–145]	0.027
PPPY COPD Hosp. Year 1	0.04	0.12	<0.001	0.65	0.38	0.004
PPPY OCS only exbs Y1	0.04	0.05	0.218	0.38	0.29	0.218
Diabetes	11%	6%	0.024	8%	9%	0.689
Cardiovascular (excluding HTN)	33%	33%	0.904	29%	37%	0.072
Reflux/heartburn	23%	23%	0.890	32%	33%	0.888
Anxiety	13%	20%	0.003	24%	16%	0.022
Depression symptoms	20%	29%	0.004	36%	28%	0.058

P-values are comparisons between those who stayed in or changed exacerbation category. Data are mean (SD) or median [25% - 75% percentile].

A total of 116 patients (6.4%) either started or stopped any maintenance treatment for COPD, i.e. LAMA, LABA, ICS and ICS/LABA combination between baseline and year 1 of follow-up; ninety-five patients (5.2%) initiated maintenance treatment and 21 (1.2%) stopped maintenance treatment.

### Change in patients characteristics over year 1

Table 3 shows the “longitudinal” changes in patient characteristics from baseline to the Year 1 assessment that took place before the category transitions. Falls in platelet count were significantly associated with a future category change from FE to IE whereas; falls in FEV_1_ and in 6MWD were significantly associated with changing from IE to FE. Table 3 also reports differences in the number of exacerbations either hospitalised and/or treated with oral corticosteroids. These two characteristics were assessed over the same time periods as the exacerbation categories and cannot therefore be considered as prospective markers. The patients who changed from IE in year 1 to FE in year 2 had 0.57 hospitalized exacerbation per patient year compared to 0.07 per patient year in IE patients who did not change category. Similarly, there were only 0.14 hospitalised exacerbations per patient year in those changing from FE to IE compared to 0.68 in those who remained FE. These results were consistent with the data on oral corticosteroid treated exacerbations.

**Table 3:** Change in COPD patient characteristics between baseline and year 1 assessment

**Year 1 Category**	**Year 1 IE** (**n**=**1289**)			**Year 1 FE** (**n**=**543**)		
**Year 2 Category**	**Stayed as IE** (**n**=**1068**)	**Change to FE** (**n**=**221**)	**p**-**value**	**Stayed as FE** (**n**=**333**)	**Change to IE** (**n**=**210**)	**p**-**value**
**Change in patient measures** (**year 1 assessment** – **baseline**)						
BMI (kg/m2)	0.0 (1.4)	0.0 (1.1)	0.750	-0.1 (1.4)	-0.1 (1.6)	0.589
FEV1 (mL)	14 (233)	-46 (184)	<0.001	-32 (180)	-22 (217)	0.543
FEV1 % predicted	0.4 (7.7)	-1.6 (6.9)	<0.001	-1.1 (6.4)	-0.9 (7.8)	0.690
FEV1/FVC %	-0.1 (5.8)	-0.4 (5.2)	0.534	-0.3 (6.0)	-0.9 (6.0)	0.250
*Changed smoking status	6%	9%	0.053	6%	9%	0.153
*Re-Started smoking (%)	5%	9%	0.051	4%	4%	0.726
*Stopped smoking (%)	7%	10%	0.448	9%	17%	0.125
6-min walk distance (m)	10 (90)	-5 (85)	0.036	-6 (94)	-1 (84)	0.568
Blood oxygen saturation (%)	0.0 (2.9)	-0.1 (3.0)	0.478	-0.3 (3.3)	-0.2 (2.8)	0.713
mMRC Dyspnoea Score	0.0 (0.9)	0.0 (1.0)	0.682	0.1 (1.0)	0.0 (1.0)	0.691
SGRQ Total Score	--2.0 (10.6)	--1.9 (12.1)	0.879	-0.1 (9.9)	-0.1 (10.2)	0.986
BODE Index	0.0 (1.2)	0.1 (1.3)	0.138	0.2 (1.5)	0.1 (1.3)	0.424
White blood cells (10^9/L)	-0.3 (1.6)	-0.2 (1.9)	0.606	0.0 (2.3)	-0.2 (2.1)	0.289
Neutrophils (10^9/L)	-0.2 (1.5)	-0.2 (1.9)	0.894	0.2 (2.3)	-0.1 (2.0)	0.232
Platelets (10^9/L)	-7 (47)	-11 (41)	0.238	-5 (51)	-16 (49)	0.014
Fibrinogen (mg/dL)	-6 [-51, 36]	0 [-38, 41]	0.151	5 [-41,58]	-3 [-52, 38]	0.109
CRP (mg/dL)	0.1 [-1.3, 1.5]	0.0 [-1.8, 2.1]	0.999	0.3 [-1.4,3.0]	-0.2 [-2.4, 1.9]	0.083
SP-D (ng/mL)	0 [-18, 20]	0 [-23, 26]	0.654	-1 [-24, 24]	0 [-21, 21]	0.806
PARC/CCL-18 (ng/mL)	6 [-6, 21]	6 [-9, 23]	0.628	9 [-6, 27]	6 [-7, 21]	0.114
Change between the number of events in year 1 and in year 2						
PPPY COPD Hosp. Year 2	0.07	0.57	<0.001	0.68	0.14	<0.001
PPPY OCS only exbs Y2	0.04	0.25	<0.001	0.37	0.06	<0.001

P-values are comparisons between those who stayed in or changed exacerbation category. Mean (SD) or Median [25% - 75% percentile].

* - “Stopped smoking” are the current smokers at baseline who stopped before Year 1, “Re-started smoking” are the former smokers at baseline who started again before Year 1.

## Discussion

This study is the first to examine in a large dataset the factors associated with changes in the exacerbation frequency category of patients with COPD. We had hoped to identify a parameter that might prospectively alert physicians to an imminent change in category but no single parameter emerged. Those variables that did change in the year before IE patients became frequent exacerbators (falls in FEV_1_ and 6-minute walking distance) or FE patients switched to IE (fall in platelet count) were too small in magnitude relative to the overall variability to provide a useful predictive tool. We did observe more hospitalized exacerbations in those changing from IE to FE, and fewer hospitalized exacerbations in those changing from FE to IE, compared to those patients who remained in their category. Since, the number of hospitalized exacerbation over year 1 partly determined how the patients were categorized; this parameter could not be considered sufficiently independent or prospective for predicting a change in category. However, it is possible that the presence, or absence, of exacerbations severe enough to warrant hospital admission indicates a transition in the trajectory of the disease. Our results highlight the multi-facetted nature of the disease and suggests that all patients are at risk of developing the frequent exacerbator phenotype.

Across the whole study population, exacerbation frequency was unchanged in year 2 compared to year 1. This agrees with another observational study that found no change in exacerbation frequency over 6 years of follow-up albeit with a symptomatic definition of exacerbation [20]. Aaron and colleagues have reported with transitional regression models that patients experience a significant acceleration in the rate of exacerbations over just one year [21]. It might be that changes in inter-exacerbation intervals provide a more sensitive measure of changes in exacerbation frequency over time, but this metric lacks the simplicity required for clinical assessment. We observed that 44% of patients had no change in exacerbation frequency between years 1 and 2. In the remaining 56% of patients, the changes in frequency had a bell-shaped distribution with an approximately equal number of rises and falls. This could simply be “regression to the mean” but there will also be some random variability in the number of viruses and bacteria circulating in the community which are thought to trigger the majority of COPD exacerbations [22,23].

The rise in exacerbation frequency that we observed in the IE group could involve four factors. Firstly, our definition of IE as 0 or 1 exacerbation per a year meant that falls in exacerbation frequency were restricted to a maximum of -1 per year, but there was no limit to any rise in frequency, and the maximum increase we observed was +10 per year. Secondly, COPD progresses and higher exacerbation frequencies are more likely to be reported in more severe patients [24]. We found that the patients who changed from IE to FE were generally more severe than those who stayed in the IE group over a wide range of patient characteristics. Thirdly, some patient characteristics fell over the year preceding the change from IE to FE. An acute deterioration is occasionally observed in clinical practice but the underlying reasons for the change are not totally recognised. The physiological markers that worsened relative to the normal reduction observed in those patients who did not change category were FEV_1_ and 6MWD. These are important indicators of COPD severity, with rapid decline in both FEV_1_[25] and 6MWD [26] associated with increased mortality. Unfortunately, the magnitude of the falls in FEV_1_ and 6MWD were small compared to their overall variability and thus would not make useful targets for monitoring risk or early intervention. Fourthly, it is possible that exacerbation frequency changed because patients started to seek treatment for previously unreported exacerbations or increased their adherence to already prescribed treatments. About 50% of exacerbations in patients with moderate to severe COPD are unreported [3]. Unreported exacerbations are generally similar to reported exacerbations in symptoms and spirometric changes [27] and contribute to a poorer quality of life [28,29] but there is much heterogeneity of reporting behaviour and even some patients with severe airflow obstruction will not report exacerbations [30].

We observed that a significant falls in platelet count occurred over the year proceeding patients changing from the FE to IE group but the standard deviation of these changes was too large to be of clinical utility. Platelet count has been found to be elevated in COPD compared to healthy controls [31] and increases during lower respiratory tract infection [32]. Platelets are not only involved in haemostasis but produce a broad array of inflammatory mediators and factors that alter innate and adaptive immunity [33,34]. We speculate that a reduced platelet count is indicative of reduced airway inflammation or improvement in immunity to infection thus reducing susceptibility to future exacerbation. There is also emerging evidence that statins, which reduce platelet-activation [35], have the potential to reduce exacerbation frequency in COPD [36].

The relatively large proportion of patients changing from FE to IE could also be explained by the method by which exacerbation frequency was assessed. A number of patients could be experiencing three exacerbations over a two year period, with for example, two exacerbations in year 1 and then one exacerbation in year 2. They would initially be categorized as FE but change to IE in the following year. Another possibility is that that patients who normally experience two exacerbations per year change to having 3 in one year and 1 in the next year, because they have an exacerbation just before, rather than just after, the division between years. Another explanation is that participation in an observational study reduces anxiety and/or improving adherence to treatment with a consequential fall in exacerbation frequency.

There are a number of limitations concerning the major findings that should be mentioned. A practice 6MWD test was not performed and approximately 6% of the results might have differed if this rehearsal had this been undertaken [37]. The declines observed in FEV_1_ in the Eclipse study [38] were modest but in-line with a recent interventional study [39]. Measurements of FEV_1_ and blood cell counts may have affected by exacerbations that took longer than 30 days to resolved, by the development of co-morbidities or initiation of pulmonary rehabilitation. The analysis was based on change in the total number of exacerbations and we did not examine the data by change in the number of hospitalized or oral corticosteroid treated exacerbations.

Amongst the strengths of this study was the follow-up of a large number of COPD patients with systematic measurements on a wide variety of patient characteristics. The patients were recruited worldwide from primary and secondary care and thus the results are generalizable to clinical practice in COPD. We did not feel that changes in oral or inhaled medication were responsible for a change in exacerbation phenotype. Our reasons were the very small proportion of patients involved and the impossibility to determining whether any change in treatment was in response to a rise in exacerbation frequency or to pre-empt an increase.

## Conclusion

Exacerbation frequency has been shown to be an important phenotype in COPD. A few patients may shift from one category to another. There is no clear explanation or marker apart from the fact that patients on the borderline between infrequent and frequent exacerbations are more likely to change category. Thus, physicians need to consider that all patients are at risk during the course of the disease of becoming a frequent exacerbator.

## Abbreviations

BMI: Body Mass Index; ICSL: Inhaled corticosteroid and long-acting b2-agonist combination; LAMA: Long acting muscarinic antagonist; ICS: Inhaled corticosteroid; LABA: Long-acting b2-agonist; OCS: Oral corticosteroid; HTN: Hypertension; HU: Hounsfield units; Hosp: Hospitalized exacerbation; PPPY: Per patient per year.

## Competing interests

GlaxoSmithKline funded the ECLIPSE study. The authors conceived and planned the current analyses and were responsible for decisions with regard to publication. The study sponsor did not place any restrictions with regard to statements made in the final paper. GD has had support from GSK to attend Eclipse meetings; HM and NL are employees of GSK; JH has had support from GSK to attend Eclipse meetings and received payments for lectures; PC had had funding from GSK for the Eclipse study; meetings; membership of the scientific committees of TORCH, ECLIPSE and SUMMIT and for lectures; JV has received funding from GSK for consultancy for the COPD phase 2&3 program and for lectures, his wife previously worked for GSK; AA has received funding from GSK for consultancy, honoraria and lectures; JW has had funding from GSK for lectures, grants and board membership. The authors have all received funding from other pharmaceutical companies.

## Authors’ contributions

Study idea and design: HM, GD, JW. Statistical Analysis: HM, NL. Interpretation of results: GC, HM, NL, JH, PC, JV, AA, JW. Manuscript drafting/ revision: GC, HM, JH, PC, JV, AA, JW. All authors read and approved the final manuscript.
